# A consensus approach to vertebrate *de novo* transcriptome assembly from RNA-seq data: assembly of the duck (*Anas platyrhynchos*) transcriptome

**DOI:** 10.3389/fgene.2014.00190

**Published:** 2014-06-25

**Authors:** Joanna Moreton, Stephen P. Dunham, Richard D. Emes

**Affiliations:** ^1^Advanced Data Analysis Centre, University of NottinghamLeicestershire, UK; ^2^School of Veterinary Medicine and Science, University of NottinghamLeicestershire, UK

**Keywords:** RNA-seq, *de novo* transcriptome, assembly, Illumina, high-throughput sequencing

## Abstract

For vertebrate organisms where a reference genome is not available, *de novo* transcriptome assembly enables a cost effective insight into the identification of tissue specific or differentially expressed genes and variation of the coding part of the genome. However, since there are a number of different tools and parameters that can be used to reconstruct transcripts, it is difficult to determine an optimal method. Here we suggest a pipeline based on (1) assessing the performance of three different assembly tools (2) using both single and multiple *k*-mer (MK) approaches (3) examining the influence of the number of reads used in the assembly (4) merging assemblies from different tools. We use an example dataset from the vertebrate *Anas platyrhynchos domestica* (Pekin duck). We find that taking a subset of data enables a robust assembly to be produced by multiple methods without the need for very high memory capacity. The use of reads mapped back to transcripts (RMBT) and CEGMA (Core Eukaryotic Genes Mapping Approach) provides useful metrics to determine the completeness of assembly obtained. For this dataset the use of MK in the assembly generated a more complete assembly as measured by greater number of RMBT and CEGMA score. Merged single *k*-mer assemblies are generally smaller but consist of longer transcripts, suggesting an assembly consisting of fewer fragmented transcripts. We suggest that the use of a subset of reads during assembly allows the relatively rapid investigation of assembly characteristics and can guide the user to the most appropriate transcriptome for particular downstream use. Transcriptomes generated by the compared assembly methods and the final merged assembly are freely available for download at http://dx.doi.org/10.6084/m9.figshare.1032613.

## Introduction

In recent years, RNA sequencing (RNA-seq) has been used to study the transcriptomic profile of many organisms. The most often used approach is to align the obtained short sequence reads to a reference genome sequence. However, when a reference is not available *de novo* transcriptome assembly can be used instead. Software pipelines to conduct this task have been developed, for example; ABySS (Simpson et al., 2009), CLC (www.clcbio.com), MIRA (Chevreux et al., 2004), Newbler (Roche), SOAPdenovo (Li et al., 2010), Trinity (Grabherr et al., 2011), and Velvet-Oases (hereafter referred to as Oases) (Schulz et al., 2012).

In *de novo* assembly transcripts are constructed by attempting to overlap reads into a contiguous sequence (“contig”), each representing a unique transcript. Unlike genome assembly where approximately even coverage (number of reads aligned at a single position) is expected, transcriptome assembly is complicated by variable coverage caused by differences in gene expression. An important parameter used in the assembly is the length of the overlapping piece of reads to join them together in an assembly, known as *k*-mer length. Robertson et al. (2010) have shown that lower *k*-mer values tend to represent lowly expressed transcripts more effectively whilst transcripts with higher coverage are better assembled with higher *k*-mer values. A multiple *k*-mer (MK) approach can therefore be adopted to capture transcripts at a wider range of expression levels compared to using a single *k*-mer (SK) assembly (Robertson et al., 2010; Surget-Groba and Montoya-Burgos, 2010; Zhao et al., 2011). In the MK strategy, assemblies generated from different single *k*-mer lengths are merged to produce a robust transcriptome of sequences expressed at different levels. The second stage of the Oases pipeline (Oases-M) was developed for this purpose (Schulz et al., 2012) as was the *de novo* transcriptome assembler Trans-ABySS (Robertson et al., 2010).

Since there are a number of different tools and parameters that can be used to reconstruct transcripts, it can be difficult to determine a single robust method. A few studies have assessed different *de novo* approaches from varying sequencing platforms and transcriptome data sets. For example, parasitic nematode 454 data (Kumar and Blaxter, 2010); simulated human 454 sequences (Mundry et al., 2012); plant paired-end Illumina data (Barrero et al., 2011) and paired-end Illumina fly, yeast and plant data sets (Zhao et al., 2011). Two of these papers focused on comparing different tools (Kumar and Blaxter, 2010; Mundry et al., 2012). Barrero et al. (2011) went on to optimize the *k*-mer value after selecting Oases from six preliminary assemblers. Zhao et al. (2011) assessed different assemblers and identified the MK approach as a significant improvement to the SK strategy. Alongside the comparison of programs, Kumar and Blaxter (2010) also merged assemblies from different assemblers and found that these generated a more credible final transcriptome. Here, we develop a pipeline to incorporate sequences from multiple assemblers and parameters to generate a robust consensus transcriptome.

Sequencing depth is also an important consideration for transcriptome assembly. Recently Francis et al. (2013) suggested that representative *de novo* assemblies can be generated from a random sub-sample of reads to achieve transcriptomes with a good balance of coverage and noise. Therefore, in our pipeline, alongside comparison of tools and parameters, we also examine the influence of the number of reads on transcriptome assembly.

## Materials and methods

### Library preparation and sequencing

Total RNA from *Anas platyrhynchos domestica* embryo fibroblasts grown in tissue culture was provided to Source Bioscience (Nottingham, UK) who carried out the library preparation and sequencing. The libraries were prepared using the Illumina TruSeq RNA Sample Preparation kit. The mRNA in the total RNA was purified using poly-T oligo-attached magnetic beads to pull down the poly-A mRNA. After purification, the mRNA was fragmented and copied into first strand cDNA using reverse transcriptase and random primers. This was followed by second strand cDNA synthesis using DNA Polymerase I and RNase H. The newly formed cDNA goes through a process of end repair, the addition of a single 'A' base and the ligation of the adapters. The samples that contain the adapters are selectively enriched for using PCR to create the final library. The libraries were validated using the Agilent BioAnalyser 2100. The libraries were clustered on to a HiSeq v3 flow cell using the Illumina cBot and sequenced on the Illumina HiSeq 2000 using a 100 base pair (bp) sequencing run generating 412 million paired-end reads. Sequence reads used in this assembly are available at European Nucleotide Archive under the study identifier PRJEB6385.

### Quality filter reads

In comparison to traditional Sanger sequencing, high-throughput sequencing is more error-prone and therefore it is important to pre-process the reads by performing quality trimming (MacManes, 2014). It is also possible for adapter fragments to remain in the read sequences and these should be removed before any downstream analysis is carried out (Lindgreen, 2012). There are many programs available for these tasks such as AdapterRemoval (Lindgreen, 2012), Cutadapt (Martin, 2011), and Trimmomatic (Lohse et al., 2012). For this study we used CLC Genomics Workbench (Version 6, www.clcbio.com) to apply quality and adapter trimming to the read sequences using the following settings: (1) Removal of low quality sequence, limit = 0.05 (2) maximal 2 ambiguous nucleotides allowed (3) minimum length 20 nucleotides. In CLC each quality score is converted to an error probability where low values represent high quality bases. For each base the error probability is subtracted from the limit (0.05 here). The cumulative total of this value (limit—error) is calculated for each base and it is set at zero if it becomes negative. The retained part of the read will start at the first positive value and end at the highest value of the cumulative total. Duplicate reads were also removed using CLC Genomics Workbench and the reads were kept if they were greater than 50 bp. Table 1 shows the effect of trimming, duplicate read removal and filtering on the number of reads.

**Table 1:** **The effect of trimming, duplicate read removal, and filtering on the number of reads**.

	**Raw**	**Trimmed**	**After duplicate removal**	
			**All**	**>50 bp**
Sequences in pairs	411,488,930	403,760,298	287,704,384	274,607,074
Orphans	0	3,592,413	2,944,998	2,393,609
Sum			290,649,382	277,000,683

*The number of reads is shown at each stage (bp, base pair)*.

### *De novo* assembly using all reads (single *k*-mers)

Velvet (version 1.2.08) (Zerbino and Birney, 2008) followed by Oases (version 0.2.08) (Schulz et al., 2012) was used to *de novo* assemble all 277 million quality filtered reads (Table 1) using odd numbers between 21 and 79 inclusively as *k*-mer values. The parameters used with Velvet were “-short, -shortPaired” and Oases “-ins_length 305, -min_trans_lgth 200” to set the minimum sequence length in the output files to 200 bp. Unfortunately the Oases assemblies for *k* = 21–31 failed likely due to a lack of memory even when running on an Ubuntu server with 24 cores (Xeon X5690, 3.46 GHz) and 192 G (1333 MHz ECC) of memory highlighting the difficulty of using relatively large data sets with all suggested options of Oases.

The CLC *de novo* assembly tool was run on all of the reads using *k* = 25 (automatic word size), *k* = 34 and *k* = 62 all with the following parameters: (1) Mapping mode = Create simple contig sequences (2) Automatic bubble size = Yes (3) Minimum contig length = 200 (4) Perform scaffolding = Yes (5) Auto-detect paired distances = Yes. Fewer *k*-mer values were used because CLC could not be set to generate assemblies for many *k*-mers in batch mode. The Oases and CLC assemblies of all 277 million reads were performed on an Ubuntu server with 24 cores (Xeon X5690, 3.46 GHz) and 192 G (1333 MHz ECC) of memory.

### *De novo* assembly using a sub-sample of reads (single *k*-mers)

Reads were subsampled using a perl script utilizing the rand function to choose “random” reads without replacement (script available at https://github.com/ADAC-UoN/subset.fastq). Velvet (version 1.2.09) followed by Oases (version 0.2.08) and ABySS (version 1.3.5) (Simpson et al., 2009) were run on a sub-sample of 30 million post-quality filtered paired reads using odd values between *k* = 21 and 79 inclusively. The “-shortPaired” parameter was used with Velvet and for Oases “-ins_length 305” and “-min_trans_lgth 200.” Default ABySS parameters were used with number of threads set as 32. The Velvet-Oases and ABySS assemblies of the sub-sample were performed on a CentOS server with 32 cores (AMD Opteron 6386SE, 2.8 GHz) and 192 G (1600 MHz DDR3 SDRAM) of memory.

The CLC *de novo* assembly tool was also run on the random sub-sample for every other odd *k*-mer value from *k* = 21 up to *k* = 63 (CLC maximum *k* = 64). The same parameters were used as the assemblies of all the reads and all CLC assemblies were conducted on an Ubuntu server with 24 cores (Xeon X5690, 3.46 GHz) and 192 G (1333 MHz ECC) of memory.

### *De novo* assembly (multiple *k*-mers)

The results from all SK assemblies were merged for each tool using the supplied Oases python script “oases_pipeline.py” (Oases-M). The default value of *k* = 27 was used for the merge as recommended in the Oases manual. For SK assemblies using all of the reads, Oases MK was run using odd values between *k* = 33 and 79 inclusively (*k* = 21–31 failed for SK) and CLC MK was run on *k* = 25, *k* = 34 and *k* = 62. These MK assemblies were executed on an Ubuntu server with 24 cores (Xeon X5690, 3.46 GHz) and 192 G (1333 MHz ECC) of memory.

For the assemblies of the sub-sample, Oases and ABySS MK were run using odd values between *k* = 21 and 79 inclusively whereas CLC MK was run on every other odd *k*-mer value in the same range. The MK assemblies of the SK sub-sample assemblies were completed on a CentOS server with 32 cores (AMD Opteron 6386SE, 2.8 GHz) and 192 G (1600 MHz DDR3 SDRAM) of memory.

### Remove redundancy and short transcript sequences

In each assembly, shorter transcripts that shared more than 99% identity with other transcripts (within a single assembly) were removed using the cd-hit-est program (Version 4.6) (Li and Godzik, 2006). Non-redundant sequences that were greater than 200 bp were kept.

### Reads mapped back to transcripts (RMBT)

To assess the validity of each of the assemblies, the reads unselected in the random sub-sampling process were aligned back to the transcript sequences using Bowtie2 (Version 2.1.0) (Langmead and Salzberg, 2012) end-to-end mode. For the assemblies generated using all of the reads, the entire set of reads was mapped back using Bowtie2 end-to-end.

### Merging assemblies for improved reliability

For comparison, one SK assembly was selected for each tool by maximizing the N50 value whilst keeping the total assembly length as long as possible (Zerbino, 2010). CAP3 (Huang and Madan, 1999) was used in an attempt to merge the three selected SK assemblies produced from the sub-sample of 30 million pairs of reads (Oases *k* = 23, ABySS *k* = 35 and CLC *k* = 25) plus the three MK sub-sample assemblies (Table 2B). Secondly, a consensus assembly was generated with CAP3 from just the three selected SK sub-sample assemblies (Oases *k* = 23, ABySS *k* = 35 and CLC *k* = 25). A final consensus assembly was created with the three selected SK sub-sample assemblies plus the assemblies produced from three largest *k*-mers (*k* = 79 for Oases and ABySS and *k* = 61 for CLC) using CAP3. The default CAP3 (VersionDate: 12/21/07) settings were used for all of the assemblies as described previously (Kumar and Blaxter, 2010). Supplemental Figure 1 shows a workflow of assembly procedure.

**Table 2:** **Assembly statistics for all reads and sub-sample of reads**.

**Assembly**	***k*-mer**	**No. transcripts**	**Total Mbp**	**Mean transcript length (bp)**	**N50 (bp)**	**RMBT (%)**	**No. transcripts >1 kb**	**CEGMA complete/partial (%)**
**(A) ALL READS: 138 MILLION PAIRS**								
Oases SK	39	153,729	255	1662	3659	92	64,243	n/d
Oases MK		1,208,328	2601	2153	3487	96	763,694	n/d
CLC SK	25	220,829	145	657	877	86	32,545	n/d
CLC MK		201,432	210	1042	1707	95	62,038	n/d
**(B) SUB-SAMPLE OF READS: 30 MILLION PAIRS**								
Oases SK	23	78,640	125	1588	3144	90	34,850	87.5/98.8
Oases MK		507,954	1014	1996	3068	94	319,913	92.7/99.6
CLC SK	25	97,375	76	781	1346	87	16,121	79.8/94.0
CLC MK		144,789	190	1315	2635	95	51,516	94.8/99.2
ABySS SK	35	53,368	47	878	1439	59	12,936	33.1/65.3
ABySS MK		89,457	108	1204	2158	87	32,797	83.5/96.8

*Abbreviations: bp, base pair; Mbp, megabase pair; kbp, kilobase pair; MK, multiple k-mer; SK, single k-mer; RMBT, reads mapped back to transcripts. Only non-redundant contigs >200 bp were assessed. CEGMA, percentage of complete and partial conserved genes identified using the CEGMA tool. **(A)** Oases MK was run using odd values between k = 33 and 79 inclusively (k = 21–31 failed). CLC MK was run on k = 25, k = 34, and k = 62. Selected SK values: Oases k = 39 and CLC k = 25. **(B)** Oases and ABySS MK were run using odd values between k = 21 and 79 inclusively whereas CLC MK was run on every other odd k-mer value in the same range. Selected SK values (based on maximizing the N50 value whilst keeping the total assembly length as long as possible): Oases k = 23, CLC k = 25, and ABySS k = 35*.

### CEGMA (core eukaryotic genes mapping approach)

As a proxy to assess the completeness of the transcriptomes assembled, the Core Eukaryotic Genes Mapping Approach (CEGMA) tool was used (Parra et al., 2007). CEGMA facilitates alignment of hidden Markov models (HMMs) of 458 core genes predicted to be ubiquitous in eukaryote species to report if a transcriptome contains predicted transcripts encoding these essential genes. The resulting completeness report details the percentage of the core genes that are either complete or partial (fragmented or truncated alignment) in the dataset.

## Results

### Generation of different *de novo* transcript assemblies

*De novo* transcriptomes were first generated using all of the 277 million quality filtered reads then on a random sub-sample of 30 million paired reads to try to establish a good balance of coverage and noise (Francis et al., 2013). The sub-sample was randomly selected from the quality filtered reads (after trimming, duplicate removal, and removal of reads less than 50 bp). A range of tools and *k*-mer values were tested for the *de novo* assemblies. Zerbino (2010) suggested using *k*-mer lengths between 21 bp and the average read length (here 89 bp) minus 10 bp. Initially Velvet-Oases and CLC Genomics Workbench were used for the assemblies of all reads. ABySS was also applied for the assembly of the sub-sample because it is less resource intensive whilst maintaining the quality of the assembly (Zhao et al., 2011). Both single and MK methods were used. The MK approach allowed the combining of lower and higher values of *k* which produce more sensitive and specific assemblies respectively (Schulz et al., 2012).

### Comparison of assemblies

**Figure 1:**
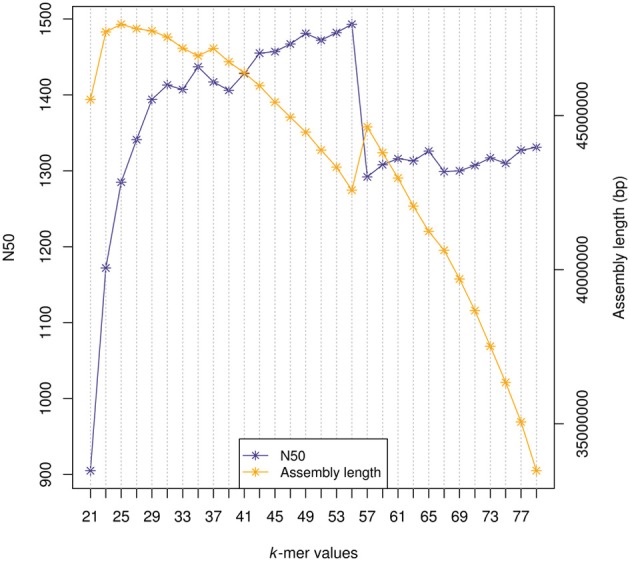
**N50 values and assembly length in base pairs (bp) for every ABySS *k*-mer assembly generated from a random sub-sample of 30 million post-quality filtered reads**.

The following metrics were assessed for each assembly: (1) number of contigs (transcripts) assembled; (2) total number of bps in the assembly; (3) Mean transcript length; (4) N50 value; (5) reads that could be mapped back to assembled transcripts (RMBT) (6) number of long transcripts (>1 kb) and (7) complete and partial core genes identified by CEGMA. For comparison, one SK assembly was selected for each tool by maximizing the N50 value whilst keeping the total assembly length as long as possible (Zerbino, 2010). The selected SK values for assemblies of all reads were Oases *k* = 39 and CLC *k* = 25 and for the sub-sample: Oases *k* = 23, CLC *k* = 25 and ABySS *k* = 35. For example, Figure 1 shows the N50 values and assembly length for all ABySS *k*-mer assemblies (30 M reads). With increased *k*-mer the N50 increases to *k* = 55 at which point the N50 deteriorates, likely due to the *k*-mer exceeding half the length of the sequence reads (average length 89 bp). The number of RMBT and CEGMA complete gene percentages are higher in the MK methods compared to SK. In particular the ABySS SK method has lower CEGMA and RMBT scores. Using these metrics the CLC MK assembly scored well with highest RMBT (95.06%) and CEGMA (94.8% complete 99.2% partial assembly of core genes). The selected SK and MK assembly statistics for all reads and the sub-sample of reads are shown in Table 2. The statistics are based on non-redundant sequences greater than 200 bp (Materials and Methods).

### Merging assemblies to provide a consensus transcriptome

In an attempt to produce a consensus assembly, CAP3 was used to merge the six assemblies (3 × SK and 3 × MK), produced from the sub-sample of 30 million pairs of reads (for read details see Table 2B). CAP3 failed to merge all six assemblies because it ran out of memory however the three selected SK assemblies (one from each tool) could be merged. Only robust contigs i.e., those that that were present in all original assemblies were retained (Kumar and Blaxter, 2010).

By using only the SK assemblies the advantage of the MK assemblies was lost by missing both sensitive and specific assemblies. The three SK assemblies that were merged had quite low *k*-mer values (Oases *k* = 23, ABySS *k* = 35 and CLC *k* = 25). Therefore, the largest *k*-mers that the assemblies were run on (*k* = 79 for Oases and ABySS and *k* = 61 for CLC) were also used in the merge to supplement the *k*-mers already selected. These six SK assemblies (two single *k*-mer assemblies from each tool) were merged with CAP3 and robust contigs for this data set were defined as those assembled by three different tools but not necessarily from all six assemblies. The merged assembly statistics (3 SK and 6 SK) are shown in Table 3 and are based on non-redundant sequences greater than 200 bp (Materials and Methods). Transcriptomes generated by the compared assembly methods and the final robust merged assembly are freely available for download at http://dx.doi.org/10.6084/m9.figshare.1032613.

**Table 3:** **Merged assembly statistics**.

**Merged assembly**	**No. CAP3 contigs**	**No. robust contigs**	**Total Mbp**	**Mean transcript length (bp)**	**N50 (bp)**	**RMBT (%)**	**No. transcripts >1 kb**	**CEGMA complete/partial (%)**
3 SK	48,302	25,573	63	2463	4006	77	17,101	79.8/88.7
6 SK	40,805	24,834	67	2689	4155	84	18,104	81.9/89.1

*Abbreviations: bp, base pair; Mbp, megabase pair; kbp, kilobase pair; SK, single k-mer; RMBT, reads mapped back to transcripts. Only non-redundant contigs >200 bp were assessed. CEGMA, percentage of complete and partial conserved genes identified using the CEGMA tool. The assemblies were generated from the sub-sample of 30 M reads. Three SK assemblies: Oases k = 23, CLC k = 25, and ABySS k = 35. Six SK assemblies: Oases k = 23 and k = 79, CLC k = 25 and k = 61, and ABySS k = 35 and k = 79. Three SK robust contigs = contigs that contained contigs from all original assemblies. Six SK robust contigs = contigs assembled by three different tools but not necessarily from all six assemblies*.

Of the final merged assemblies, the one that combined the six SK assemblies had a higher percentage of reads mapping back to transcripts (RMBT) compared to the 3 SK assembly. The CEGMA analysis revealed that the 81.9% of core genes are complete in the 6 SK assembly and 89.1% of genes are present in a partial form. The mean transcript length, N50 values and number of long transcripts (>1 kb) were also higher for the six-merged assemblies compared to the three-merged (Table 3). The RMBT percentages were generally lower for the merged assemblies compared to the individual SK and MK assemblies (Tables 2, 3). However, this was to be expected as only robust contigs were considered for the merged assembly. The N50 values increased for the merged assemblies (maximum 4155, Table 3) compared to the individual SK and MK assemblies (maximum 3659, Table 2).

## Discussion

The SK assembly metrics from the different tools varied, for example CLC was quicker but generated contigs with a lower N50 value. Among the SK assemblies, Oases produced the ones with the highest number of bps, mean transcript lengths, N50 values, RMBT percentages, and long transcripts (Table 2). This tool also took less time to assemble the transcripts compared to ABySS. It is difficult to compare the MK assemblies from the three tools directly because different numbers of *k*-mer assemblies were generated. For instance, some of the Oases assemblies (*k* = 21–31) failed from insufficient memory when all reads were used. However, in comparison to SK, the MK assemblies were longer and had a higher percentage of reads mapping back to transcripts (RMBT) which is an important measure for evaluating the assembly (Zhao et al., 2011) and a greater number of core genes identified by CEGMA, suggesting a more complete transcriptome. Together, this suggests the MK assemblies are likely to represent a wider range of transcripts.

The commonly used metrics to determine assembly quality (N50 values and RMBT percentages) show the variability between assemblies. Importantly the sub-sample of reads requires much less time was and computational power making this method more tractable for those with limited memory resources. The use of a sub-sample of reads can also provide further validation by mapping the unselected reads (those not used in the assembly) to the generated assembly. Using this approach, the RMBT percentages were lower for the merged assemblies but only robust contigs were considered so this was expected. The N50 values increased greatly for the merged assemblies compared to the individual SK and MK assemblies. Of the final merged assemblies, the one that combined the six SK assemblies had a higher RMBT percentage, larger mean transcript length, larger N50 value and more long transcripts (>1 kb) compared to the three-merged. We suggest that the lower RMBT values seen are not of concern when the aim is to generate a robust assembly that contains high quality transcripts. This value may be more relevant if the aim is to generate the most comprehensive transcriptome set. This is also true for the CEGMA analysis, where the final “robust” transcriptome (6 SK), which did not have the highest percentage of complete expected genes (81.85% compared to a maximum of 94.76% from the CLC MK assembly), however we believe the 6 SK assembly represents a more cautious assembly by reducing potential false positive transcripts. The 6 SK assembly also has a much higher proportion of longer transcripts (>1 Kb) 18,104/24,834 (72.9%). In contrast the CLC MK assembly has only 35.6% longer transcripts (51,516/144,789 see Table 2) suggesting that shorter, potentially fragmented transcripts dominate the assembly. The need to create a *de novo* assembly suggests that the “truth” is not known and hence all assemblies will necessitate a compromise to balance many different parameters. The downstream use of the assembly should be considered when selecting methods for assembly. All the assemblies generated by this study are available at http://dx.doi.org/10.6084/m9.figshare.1032613 and may be utilized by different groups for different purposes. For example if the most comprehensive transcriptome is required possibly the CLC MK assembly (highest CEGMA score, highest RMBT score) would be valid.

The results here suggest that a robust *de novo* transcriptome can be generated, with limited computational resources using (1) a random sub-sample of the reads; (2) three different assembly tools; (3) merging the assemblies of two SK assemblies from each tool. However, we stress that the user should use assembly metrics such as RMBT and CEGMA scores or similar to understand and balance the breadth (number of transcripts discovered) and robustness (completeness of the transcripts identified) for their particular needs. In our approach, reads were sub-sampled to establish a good balance of coverage and noise (Francis et al., 2013). A high proportion of reads unselected in the sub-sampling process map to transcripts generated from the sub-sample. This suggests that sub-sampling does not drastically impact on the complexity of the transcriptome generated even though more reads were used than suggested by Francis et al. (2013). For the third part of the pipeline we selected (for each tool) one SK assembly by maximizing the N50 value whilst keeping the total assembly length as long as possible (Zerbino, 2010) and secondly the largest *k*-mer that the assemblies were run on. This was to try to take advantage of the MK approach by combining more sensitive and specific assemblies (Schulz et al., 2012) without running out of memory when merging the transcriptomes. The three stage approach proposed enables the efficient use of different tools and parameters to reconstruct a robust consensus of vertebrate transcripts. The second stage resulted in a more comprehensive assembly, whereas the last stage produced an assembly with longer transcripts that was likely to have fewer false positives, but was also less comprehensive.

### Conflict of interest statement

The authors declare that the research was conducted in the absence of any commercial or financial relationships that could be construed as a potential conflict of interest.

## Supplementary material

Supplemental Figure 1 **Cartoon schematic of experimental workflow**.
