# Supplementary figures and images for: A consensus approach to vertebrate de novo transcriptome assembly from RNA-seq data: assembly of the duck (Anas platyrhynchos) transcriptome
Source: Front Genet. 2014 Jun 25;5:190. doi: 10.3389/fgene.2014.00190 (PMC4070175; doi:10.3389/fgene.2014.00190)

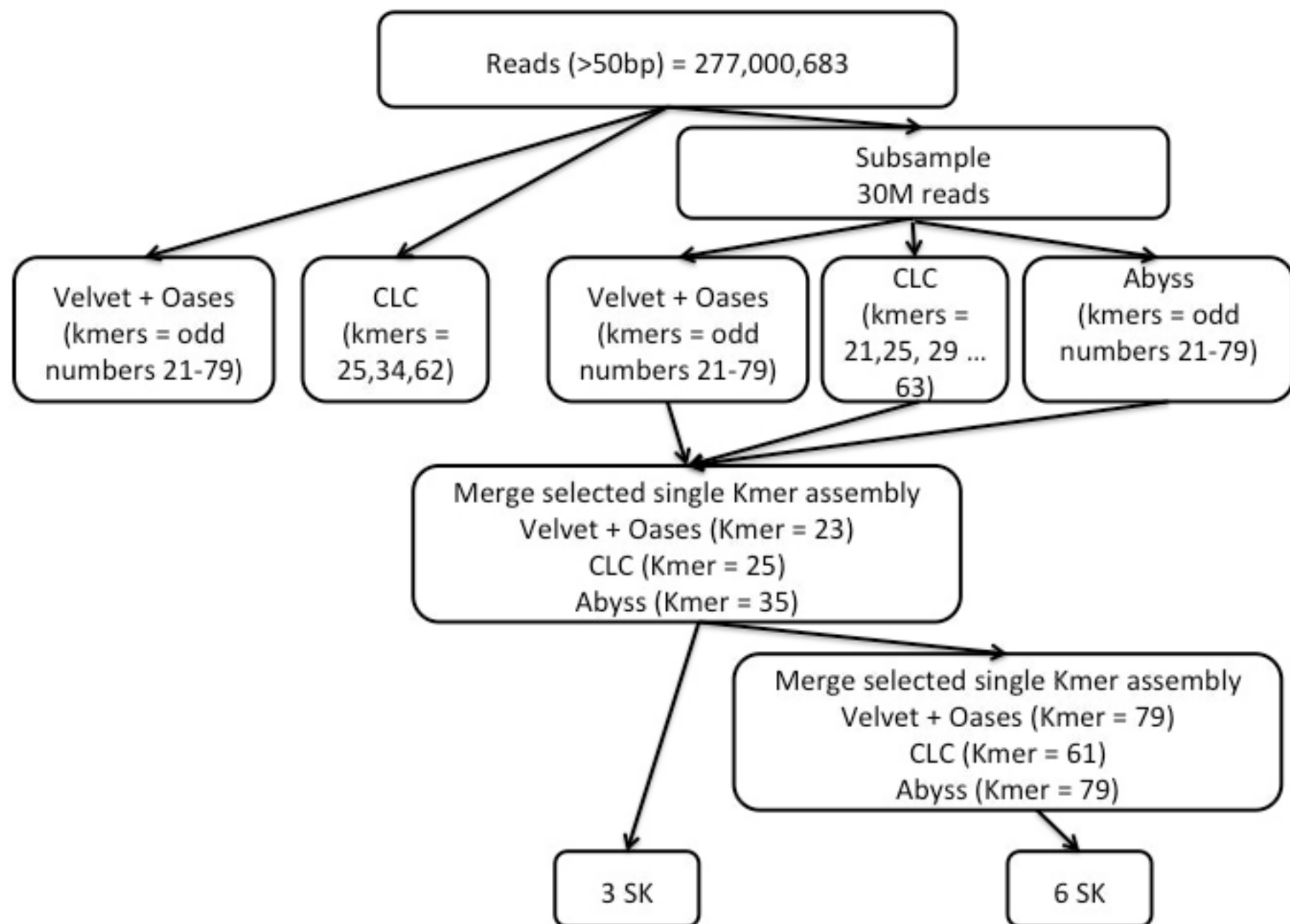

Supplemental Figure 1: Cartoon Schematic of experimental workflow.

Supplement: Supplemental Figure 1 — Cartoon schematic of experimental workflow. [file DataSheet1.PDF]
